# The influence of MRI scan position on patients with oropharyngeal cancer undergoing radical radiotherapy

**DOI:** 10.1186/1748-717X-8-129

**Published:** 2013-05-28

**Authors:** Scott Hanvey, Mark McJury, Lye Mun Tho, Martin Glegg, Maureen Thomson, Derek Grose, Allan James, Mohammed Rizwanullah, Claire Paterson, John Foster

**Affiliations:** 1 Clinical Physics, University of Glasgow, Wolfson Medical School Building, University Avenue, Glasgow G12 8QQ, Scotland, UK; 2 Department of Clinical Physics and Bioengineering, Beatson West of Scotland Cancer Centre, 1053 Great Western Road, Glasgow, Scotland G12 0YN, UK; 3 Department of Clinical Oncology, Beatson West of Scotland Cancer Centre, 1053 Great Western Road, Glasgow G12 0YN, Scotland, UK; 4 Clinical Oncology Unit, University of Malaya Cancer Research Institute, Faculty of Medicine, University of Malaya, Kuala Lumpur, Malaysia

**Keywords:** MRI, CT, Oropharyngeal, Radiotherapy, Image registration, Patient setup

## Abstract

**Background:**

The purpose of this study was to demonstrate how magnetic resonance imaging (MRI) patient position protocols influence registration quality in patients with oropharyngeal cancer undergoing radical radiotherapy and the consequences for gross tumour volume (GTV) definition and radiotherapy planning.

**Methods and materials:**

Twenty-two oropharyngeal patients underwent a computed tomography (CT), a diagnostic MRI (MRI_D_) and an MRI in the radiotherapy position within an immobilization mask (MRI_RT_). Clinicians delineated the GTV on the CT viewing the MRI_D_ separately (GTV_C_); on the CT registered to MRI_D_ (GTV_D_) and on the CT registered to MRI_RT_ (GTV_RT_). Planning target volumes (PTVs) were denoted similarly. Registration quality was assessed by measuring disparity between structures in the three set-ups. Volumetric modulated arc therapy (VMAT) radiotherapy planning was performed for PTV_C_, PTV_D_ and PTV_RT_. To determine the dose received by the reference PTV_RT_, we optimized for PTV_C_ and PTV_D_ while calculating the dose to PTV_RT_. Statistical significance was determined using the two-tailed Mann–Whitney or two-tailed paired student t-tests.

**Results:**

A significant improvement in registration accuracy was found between CT and MRI_RT_ versus the MRI_D_ measuring distances from the centre of structures (geometric mean error of 2.2 mm versus 6.6 mm). The mean GTV_C_ (44.1 cm^3^) was significantly larger than GTV_D_ (33.7 cm^3^, *p* value = 0.027) or GTV_RT_ (30.5 cm^3^, *p* value = 0.014). When optimizing the VMAT plans for PTV_C_ and investigating the mean dose to PTV_RT_ neither the dose to 99% (58.8%) nor 95% of the PTV (84.7%) were found to meet the required clinical dose constraints of 90% and 95% respectively. Similarly, when optimizing for PTV_D_ the mean dose to PTV_RT_ did not meet clinical dose constraints for 99% (14.9%) nor 95% of the PTV (66.2%). Only by optimizing for PTV_RT_ were all clinical dose constraints achieved.

**Conclusions:**

When oropharyngeal patients MRI scans are performed in the radiotherapy position there are significant improvements in CT-MR image registration, target definition and PTV dose coverage.

## Background

With intensity modulated radiotherapy and volumetric modulated arc therapy (VMAT), it is possible to deliver high doses of radiation to irregular target volumes whilst sparing normal tissue, which can result in reduced severity of radiation toxicities [1]. Increased dose delivery and dose conformity has led to a greater significance on the accurate localization of the gross tumour volume (GTV) and neighbouring structures.

In radiotherapy (RT) planning, computed tomography (CT) remains the first choice since it provides accurate dosimetric information. Imaging modalities such as magnetic resonance imaging (MRI) and positron emission tomography (PET) may present advantages compared to CT in terms of target definition. In particular, MRI offers improved soft tissue contrast [2,3] and reduced artefacts from dental amalgam; hence, MRI is the imaging modality of choice for oropharyngeal cancers [4]. MRI improves target definition for patients with head and neck [5-7], prostate [8,9] and brain [10,11] cancer.

Challenges with MRI in clinical practice include: geometric distortion [12,13]; motion related artefacts [14] and magnetic susceptibility [15,16]. Patient positioning must also be considered, since differing patient set-ups between modalities can result in uncertainties in the location and magnitude of the GTV and thus the dose received by the patient [17].

Patients receiving RT to the brain can undergo an MRI in the RT position within an immobilization mask, without loss of image quality over standard imaging methods [18]. An approximate image registration uncertainty of 2 mm occurs when registering CT image sets in the treatment position to a diagnostic MRI for patients with brain cancer [19] and therefore many RT centres routinely register these scans for RT planning. While it could be anticipated that imaging patients with oropharyngeal cancer in the treatment position would result in improved registration and target volume definition, to our knowledge there is no published evidence to suggest that using the diagnostic MRI provides results to the contrary. In addition, no publications have analyzed the dosimetric consequences of patient positioning during MRI scan acquisition.

The purpose of this study was to compare an MRI acquired with a diagnostic patient position on a standard MRI table to an MRI acquired on a flat table with custom immobilization and to determine how this affects CT-MRI registration, GTV definition, the resulting VMAT RT plans and the magnitude of the associated geometric and dosimetric errors. The results obtained would then provide an answer to whether it was necessary to obtain a planning MRI scan for patients with oropharyngeal cancer in the RT treatment position using an immobilization mask or whether a diagnostic patient position protocol would suffice.

## Methods

### Patient group and study overview

Twenty two patients with oropharyngeal cancer (age 37–72), being worked up for radical RT (patients being treated with curative intent), were identified for the study, regardless of tumour or nodal stage. The study protocol was approved by Local Ethics Committee (West of Scotland Research Ethics Service, Western Infirmary, Glasgow G11 6NT, Scotland, UK) and informed written consent was obtained from all patients. Registration quality assessment was conducted on all patients, however, in three patients a GTV evaluation was not possible, since two patients had undergone primary surgical resection and another had received induction chemotherapy, resulting in complete response.

**Figure 1:**
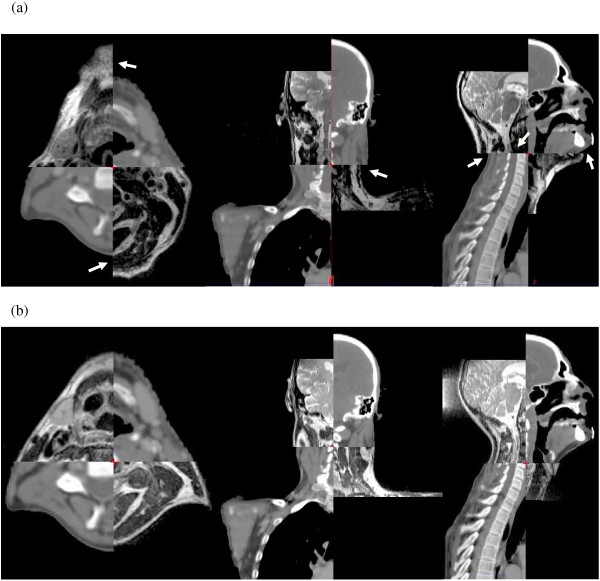
**Split view showing the registration in the axial, coronal and sagittal planes of (a) the CT and MRI**_**D **_**and (b) the CT and MRI**_**RT **_**datasets.** Arrows indicate regions where there is a registration mismatch between CT and MRI_D_. Typically, more discrepancies in registration occurred with MRI_D_ than MRI_RT_. MRI_D_: diagnostic MRI scan; MRI_RT_: radiotherapy positioned MRI scan.

Patients underwent a CT planning scan, and two MRI scans (patients scanned between February 2010 and January 2012; median time between CT and MRI scans = 5 days, range = 0 to 21 days). The first MRI scan was obtained with the patient in the standard diagnostic position (denoted as MRI_D_) and the second with the patient in an immobilization mask in the RT position (MRI_RT_). Both MRI scans were registered separately with the CT planning scan as shown in Figure 1. The GTV was delineated on the CT images by trained Consultant Radiation Oncologists using the treatment planning system, with the aid of viewing MRI_D_ on a separate console. This patient position protocol is denoted as PP_C_. This is the current standard practice at out centre, and would be considered standard practice in the majority of institutions in this country. For the purposes of this study, the GTV was delineated using two other patient set-ups. Firstly, the GTV was delineated on the MRI_D_ registered to the CT datasets. This patient position protocol is denoted as PP_D_. The GTV was also delineated on the MRI_RT_ registered to the CT image sets, denoted as PP_RT_.

### CT and MRI scanning protocol

Patients were scanned on a GE Light-speed RT 16 slice CT scanner (GE Healthcare, WI, USA), using the current clinical scanning protocol, and immobilized within a full face and neck five point fixation thermoplastic beam directional shell (BDS) with appropriate head rest (CIVCO Medical Solutions, IA, USA). The scan extent was from superior orbital ridge to carina. A helical scan was acquired with a detector configuration of 16 × 1.25, pitch 0.938, matrix 512 × 512 and speed 18.75 mm/rot with a slice thickness of 2.5 mm.

**Figure 2:**
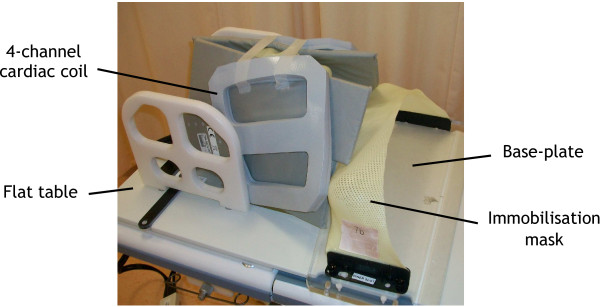
MRI acquired in the radiotherapy position with a 4-channel flexible surface coil positioned laterally.

Patients underwent MRI scans in two different positions during the same scan session. For the MRI_RT_ scan, patients were positioned on a flat MRI Oncology Table (GE Healthcare, WI, USA), within a BDS, with a 4-channel flexible surface cardiac coil positioned laterally over the patient’s neck (Figure 2). The same scan extent as the CT scan was employed and the imaging parameters, chosen in accordance with local protocol, are presented in Table 1.

**Table 1:** MRI imaging parameters

**Scan type**	FOV* (mm)	**Slice thick (mm)**	**Spacing (mm)**	**TE† (ms)**	**TR†† (ms)**	**Bandwidth (± kHz)**	**Matrix size**
2D Driven-equilibrium FSE (T2-weighted)	400	2.5	0	94	2620	63	512 × 256
3D Spoiled gradient echo (T1-weighted post-contrast)	400	2.5	0	2	15	50	256 × 256

* Field of view.

† Echo time.

†† Repetition time.

The scan was repeated for the MRI_D_ on a standard curved diagnostic table without BDS using a 16-channel head, neck and spine coil, with the CT scan extent. The 16-channel head, neck and spine coil was not used for the MRI_RT_ scan since it is not compatible with the BDS. A previous investigation using test objects has shown the image quality obtained using the 4-channel cardiac coil is of diagnostic quality [18].

### Image registration

The treatment planning system (Eclipse, Varian Medical Systems, Inc. CA, USA, version 10.0.39) was used to register the MRI_RT_ and MRI_D_ T1 and T2-weighted scans to the CT data. This software enabled fully automated, mutual-information based rigid registration to be performed. While deformable registration is available for Eclipse, it does not allow inter-modality registration. Since commercial inter-modality deformable image registration is generally not available, nor employed clinically in our centre at present, this was not subject to further investigation. The volume over which the registration was performed was centred over the oropharyngeal region and chosen to include as much information for the registration algorithm as possible [6]. Fully automated registration was used without manual adjustment since, for this site, satisfactory registration for both target and nodes is not always achievable and would introduce subjective errors.

To quantitatively assess the quality of registration the orbits and the odontoid process were outlined on the CT and T1-weighted MR image sets. These three structures were delineated on the CT, for each patient, and then repeated on the MR datasets registered to the CT.

Two metrics determined the quality of registration. Firstly, the distance between the centre of the orbits and odontoid process drawn on the CT and the MRI datasets was calculated using the coordinate location of the centre of the structures determined by the treatment planning software. The geometric mean was calculated to ensure normality of the data. Secondly, the quality of registration was assessed by measuring the spatial overlap of these structures drawn on CT and MRI. The spatial overlap was assessed by calculating the Dice coefficient (spatial overlap) for each structure, which is given by,

$$\mathrm{spatial}\;\mathrm{overlap}=\frac{\mathrm{CT}\cap \mathrm{MRI}}{\left(\mathrm{CT}+\mathrm{MRI}\right)/2}$$

where CT ∩ MRI is the volume of intersection between the CT and MRI structures. The value of the spatial overlap can range from zero, which indicates no spatial overlap between the CT and MRI volumes, to one, which indicates complete overlap [20]. Since structures outlined on CT and MRI may differ, even with perfect registration, a spatial overlap of one may not be achieved in practice, but will still be dependent on different patient set-up and registration quality.

### Gross tumour volume, lower risk clinical target volume and organ at risk delineation

Three Oncologists were assigned five patients each and a fourth was assigned four patients. The Oncologists delineated the GTV on their patients using the three set-ups PP_C_, PP_D_ and PP_RT_, which in this study are referred to as GTV_C_, GTV_D_ and GTV_RT_ respectively. Anonymized information sheets, containing the patient’s clinical history and radiology report, were available to the Oncologists. The clinicians generally utilized the T2-weighted MR datasets while contouring the GTV, although T1-weighted images were also referenced. A period of at least a week was given between delineations of the GTV for the same patient using a different imaging protocol and the Oncologists were blinded to previous delineations. Changes in the magnitude of the GTV were assessed. Contouring was also performed on the lower risk clinical target volume (CTV LR) of the nodal areas at risk of microscopic involvement for a randomly selected cohort of ten patients, in each of the set-ups. Nodal delineation was performed according to international consensus guidelines [21]. For these patients, the organs at risk (OARs), which included the left and right parotids, larynx, spinal cord and the brainstem, were also contoured.

The GTV and CTV LR were expanded to obtain planning target volumes, (PTV) and lower risk planning target volumes (PTV LR) respectively. To create the clinical target volume (CTV) the GTV was expanded by 1 cm isotropically, removing any overlap with bone or air cavities. The CTV was then enlarged by 3 mm isotropically and cropped from the external outline of the body to create the PTV. It is necessary to crop the PTV from the body outline to assist in the VMAT optimization process. The PTVs were generated from the GTVs using the three set-ups PP_C_, PP_D_ and PP_RT_, which are denoted as PTV_C_, PTV_D_ and PTV_RT_ respectively. Similarly, to generate the PTV LR the CTV LR was expanded by 3 mm isotropically and cropped by an appropriate margin from the body outline. Target volumes and OARs were generated in accordance with local protocol.

### Dose analysis

VMAT plans were calculated for the ten patients for whom the GTV, CTV LR and OARs were contoured, to determine the impact that changes in target volume definition have on RT planning. The VMAT plans were calculated in accordance with our centre’s clinical dose constraints using the Anisotropic Analytical Algorithm and Progressive Resolution Optimizer VMAT algorithm in Eclipse (Varian Medical Systems, Inc, CA, USA) version 10.0.28. A VMAT plan was calculated for PTV_C_, PTV_D_ and PTV_RT_. Dose volume histograms (DVHs) were generated for PTV_C_, PTV_D_ and PTV_RT_. A mean DVH for all ten patients was then calculated for PP_C_, PP_D_ and PP_RT_. These results are presented using our centre’s dose constraint protocol i.e. D_99_ > 90%, D_95_ > 95%, D_5_ < 105% and D_2_ < 107%, where D_99_ > 90% means 99% of the total PTV volume should receive a dose > 90% of the prescribed dose. The other dose constraints are defined similarly.

Of particular interest in this study was to establish the quality of the RT plan for PP_C_ and PP_D_ with reference to PP_RT_, since our working hypothesis postulates that the optimum target volume definition is achieved with PP_RT_. To achieve this we optimized for PTV_C_ and PTV_D_ but investigated the dose coverage of PTV_RT_ at D_99_ > 90%, D_95_ > 95%, D_5_ < 105% and D_2_ < 107%.

A quantitative comparison of the homogeneity of the dose to the PTV was completed using the sigma index. The sigma index was compared individually for PTV_C_, PTV_D_ and PTV_RT_ as well as for PTV_C_, PTV_D_ with reference to PTV_RT_. The sigma index is equal to the standard deviation of the dose throughout the PTV, calculated on a voxel by voxel basis [22], thus the higher the sigma index, the greater the dose inhomogeneity.

Finally, the dose to the OARs was assessed by evaluating the mean and maximum dose received using PP_C_, PP_D_ and PP_RT_.

Two-tailed paired student t-tests were performed to examine the statistical differences of the registration quality and dosimetric indices, except for the geometric mean distance from the centre of structures in CT and MRI where two-tailed Mann–Whitney tests were performed. A Mann–Whitney test was used to assess the geometric mean distance from the centre of the structures because the data was not normally distributed. The null hypothesis was rejected when the *p* value was less than 0.05.

## Results

**Figure 3:**
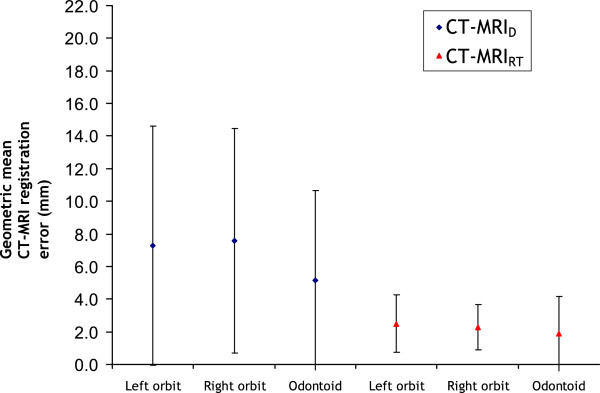
**Quality of registration results.** The geometric mean CT-MRI registration error is the geometric mean of the distance from the centre of the CT structures to the centre of the MRI structures. The error bars represent ± 1 standard deviation. Odontoid: odontoid process; CT-MRI_D_: CT registered with the diagnostic MRI scan; CT-MRI_RT_: CT registered with radiotherapy positioned MRI scan.

There was a reduction in the geometric mean distance from the centre of the orbits and odontoid process delineated on the CT and MRI_RT_ to that delineated on the CT to MRI_D_ volumes (Figure 3) and this was significant (*p* value < 0.001) for each structure. Narrower error bars exist for the CT registered to the MRI_RT_ versus the MRI_D_, which strongly implies that the patients are positioned more closely to their CT set-up in the BDS than when using the ordinary diagnostic set-up. No correlation was found between the time from the CT and MRI scan in days and the mean registration error for either MRI_D_ (R^2^ = 0.06) or MRI_RT_ (R^2^ = 0.02).

An improvement in the mean spatial overlap for the orbits and odontoid process was observed between CT and the CT-MRI_RT_ over the CT to CT-MRI_D_ data sets as shown in Table 2. Analyzing the difference between the spatial overlap of CT to CT-MRI_D_ versus CT to CT-MRI_RT_ was shown to have a *p* value < 0.001 for both orbits and odontoid process (Table 2). The volume of the three structures delineated on CT was larger than those delineated on MRI, however, their mean difference was within one standard deviation. Therefore, the spatial overlap is expected to give a good measure of the quality of registration.

**Table 2:** Mean spatial overlap of the anatomical landmarks for the two registration set-ups

	**CT-MRI**_D_* mean spatial overlap	**CT-MRI**_**RT**_**† mean spatial overlap**	***p *value**
Left orbit	0.49	0.81	< 0.001
Right orbit	0.48	0.81	< 0.001
Odontoid process	0.37	0.67	< 0.001

* CT registered with diagnostic MRI.

† CT registered with the radiotherapy positioned MRI.

The mean GTV_C_ was significantly larger than the GTV_D_ or GTV_RT_ as shown in Table 3. The *p* values in Table 3 refer to the differences in the magnitude of the GTV_C_ and the other two GTVs. There was no significant difference between GTV_D_ and GTV_RT_ (*p* value = 0.14).

**Table 3:** Mean GTV* (cm^**3**^**) delineated with the different patient position protocols**

**Mean GTV**_**C**_**†**	**Mean GTV**_**D**_**†† (*p *value)**	**Mean GTV**_**RT**_^**# **^**(*p *value)**
44.1	33.7 (0.027)	30.5 (0.014)

The *p* value relates to differences in the magnitude of GTV_C_ and the other two GTVs.

* Gross tumour volume.

† GTV delineated on CT with diagnostic MRI scan viewed separately.

†† GTV delineated using the CT registered with diagnostic MRI.

^#^ GTV delineated using the CT registered with radiotherapy positioned MRI.

**Figure 4:**
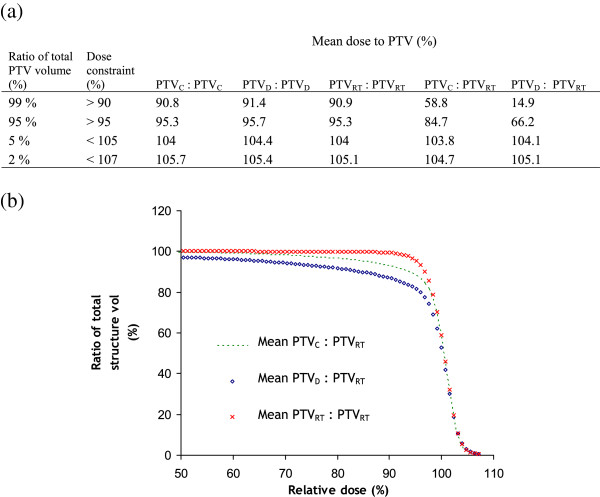
**Mean DVH values and mean DVHs.** (**a**) Values obtained from mean DVHs optimizing for each PTV but investigating the dose to each PTV and (**b**) mean DVHs optimizing for each PTV but investigating the dose to PTV_RT_. The PTV left of the colon indicates the PTV for which the VMAT plan was optimized and right of the colon indicates the PTV under examination. DVH: dose volume histogram; PTV_C_: PTV delineated using the CT with the diagnostic MRI scan viewed on a separate console; PTV_D_: PTV delineated on the CT registered with the diagnostic MRI scan; PTV_RT_: PTV delineated on the CT registered with the MRI scan in the radiotherapy position.

Clinical dose constraints were met for the mean DVHs of PTV_C_, PTV_D_ and PTV_RT_ (columns 3–5 of Figure 4(a)), which validates the planning methodology in this study. When optimizing the RT plan for PTV_C_, or repeating the optimization process for PTV_D_, the dose to PTV_RT_ shows that neither the D_99_ nor the D_95_ dose constraints are met for the mean DVHs of PP_C_ or PP_D_ (the last 2 columns of Figure 4(a) and (b)). For example, for D_99_ mean dose to the PTV for PTV_D_: PTV_RT_ is 14.9% rather than the 90% required. Conformity to dose constraints is poorer for PTV_D_: PTV_RT_ compared to PTV_C_: PTV_RT_ (Figure 4(b)). Only by using PP_RT_ can all dose constraints be achieved for PTV_RT_.

The justification for choosing only ten patients to perform the dose analysis was that none of the ten patients met the 90% dose constraint for PTV_C_: PTV_RT_ and PTV_D_: PTV_RT_ and only one of the ten patients met the 95% dose constraint for both PTV_C_: PTV_RT_ and PTV_D_: PTV_RT_. This relates to a 95% confidence interval of 0.0% to 30.9% and 0.3% to 44.5% respectively. These confidence intervals demonstrate that no further cases are statistically necessary in the analysis.

**Figure 5:**
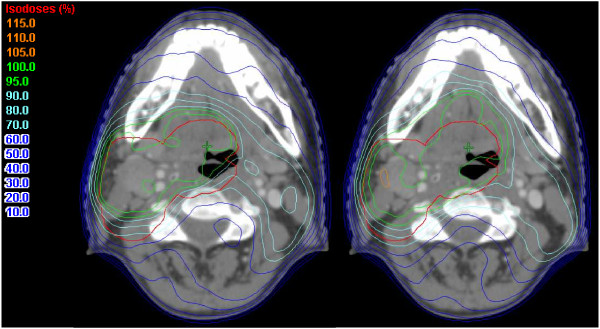
**PTV**_**RT **_**and the dose distributions optimized for PTV**_**C **_**(left) and optimized for PTV**_**D **_**(right).** PTV_RT_: PTV delineated on the CT registered with the MRI scan in the radiotherapy position; PTV_C_: PTV delineated using the CT with the diagnostic MRI scan viewed on a separate console; PTV_D_: PTV delineated on the CT registered with the diagnostic MRI scan.

Figure 5 demonstrates for a typical patient the PTV_RT_ and the dose distributions optimized for PTV_C_ (left) and PTV_D_ (right). This figure shows that the 95% isodose line does not cover PTV_RT_ entirely with a posterior proportion of the PTV receiving a dose less than 95% of the prescribed dose. Figure 5 is in agreement with the results of Figure 4 which shows that when optimizing for PTV_C_ or PTV_D_ not all the dose constraints are met for PTV_RT_.

The mean sigma indices are shown in Table 4. In the first three columns of Table 4 the sigma indices are within 3.3% again validating the planning methodology in this study. The dose homogeneity becomes considerably poorer for PTV_C_: PTV_RT_ and PTV_D_: PTV_RT_ at 7.3% and 9.1% respectively. A statistically significant difference between the sigma indices was found between PTV_C_: PTV_C_ and PTV_C_: PTV_RT_ (*p* value = 0.004) and between PTV_D_: PTV_D_ and PTV_D_: PTV_RT_ (*p* value = 0.008). The mean sigma index for PTV_D_: PTV_RT_ is poorer than for PTV_C_: PTV_RT_ in agreement with the results of Figure 4(a).

**Table 4:** Mean sigma indices for the PTVs

	**PTV**_**C**_**: PTV**_C_*	**PTV**_**D **_**PTV**_**D**_**†**	**PTV**_**RT**_**: PTV**_**RT**_**††**	**PTV**_**C**_**: PTV**_**RT**_	**PTV**_**D**_**: PTV**_**RT**_
Mean sigma index (%)	3.0	3.1	3.3	7.3	9.1

PTV left of the colon indicates the PTV for which the VMAT plan was optimized and right of the colon the PTV under examination.

* PTV delineated using the CT with diagnostic MRI viewed separately.

† PTV delineated on the CT registered with diagnostic MRI.

†† PTV delineated on the CT registered with radiotherapy positioned MRI.

Results for parotid and larynx OAR dose analyses are presented in Table 5. In situations where there was overlap between a parotid and PTV, it was considered that dose sparing to that parotid was not possible without compromising PTV dose; hence it was excluded from the analysis. The results demonstrate that in planning PTV_C_, PTV_D_ and PTV_RT_, in all 10 patients the mean dose to the parotid was < 2400 cGy which met clinical dose constraints. However, when RT plans were optimized for PTV_C_ and PTV_D_ and the dose to the parotid for PP_RT_ were analysed (PTV_C_: PTV_RT_ and PTV_D_: PTV_RT_) in 5 out of 10 patients the parotid dose exceeded the tolerance. For the larynx, the tolerance of 4000 cGy was exceeded in 4 out of 10 for PP_C_, 3 out of 10 for PP_D_ and 1 out of 10 patients for PP_RT_. However, as with the parotid, when RT plans were optimized for PTV_C_ and PTV_D_ and the dose to the larynx for PP_RT_ were analysed there were more instances of unmet dose constraints (5 out of 10 patients for both PTV_C_: PTV_RT_ and PTV_D_: PTV_RT_).

**Table 5:** Mean dose for the (a) parotid and (b) larynx

**(a)**					
	**Parotid mean dose (cGy)**				
	**Mean dose constraint 2400 cGy**				
	**PP**_**C**_**: PP**_C_*	**PP**_**D**_**: PP**_**D**_**†**	**PP**_**RT**_**: PP**_**RT**_**††**	**PP**_**C**_**: PP**_**RT**_	**PP**_**D**_**: PP**_**RT**_
Mean	2179.6	1965.5	1820.3	2233.8	1992.4
Number of patients which exceed the dose constraint	0	0	0	5	5
**(b)**					
	**Larynx mean dose (cGy)**				
	**Mean dose constraint 4000 cGy**				
	**PP**_**C**_**: PP**_**C**_	**PP**_**D**_**: PP**_**D**_	**PP**_**RT**_**: PP**_**RT**_	**PP**_**C**_**: PP**_**RT**_	**PP**_**D**_**: PP**_**RT**_
Mean	3979.6	3892.3	3613.2	4108.8	4020.6
Number of patients which exceed the dose constraint	4	3	1	5	5

Where there was overlap between a parotid and the PTV the parotid dose was not included in the mean parotid dose. PP left of the colon indicates the patient position protocol for which the VMAT plan was optimized and right of the colon indicated the patient position protocol under examination.

* Patient position protocol: CT and diagnostic MRI scan viewed separately.

† Patient position protocol: CT registered with the diagnostic MRI.

†† Patient position protocol: CT registered with radiotherapy positioned MRI.

When the mean dose to the parotid and larynx for all 10 patients was calculated, an incrementally smaller value was seen for PTV_C_, PTV_D_ and PTV_RT_ (first 3 columns of Table 5), which can be explained by the decrease in the magnitude of the GTV. Dose to the spinal cord and brainstem were also calculated but were not found to exceed clinically relevant tolerances for any of the patient position protocols.

## Discussion

Advanced imaging techniques have been shown to improve tumour and nodal staging [23] and the benefits of integrating MRI are well known [5-7,23,24]. While the advantages in positioning patients in a similar way to their CT planning scan when acquiring MR images are also known [6,17,23], this is the first study to compare the CT-MRI registration accuracy and dosimetric effects of a diagnostic versus RT positioned MRI scan in patients with oropharyngeal cancer.

During the process of registering CT to MR images, there was improved registration with fewer discrepancies using MRI_RT_ compared to MRI_D_, as demonstrated in Figure 1. In this example, there are registration discrepancies at the body outline and spinal cord for CT-MRI_D_, as indicated by the arrows (Figure 1(a)), but not with MRI_RT_ (Figure 1(b)).

A significant improvement in the registration quality of CT to MRI_RT_ versus the MRI_D_ was demonstrated by a reduction in the geometric mean distance from the centre of the orbits and odontoid process delineated on CT and the MRI_RT_ and an increase in the spatial overlap of these structures. These results show that patient setup significantly influences CT-MRI registration accuracy. With increased interest in the use of dose escalation and dose painting techniques within RT planning the importance of improved image registration becomes ever more relevant.

The significant difference between the magnitude of the GTV_C_ and GTV_D_ and between the GTV_C_ and GTV_RT_ implies that the GTV is significantly smaller when using registered rather than unregistered CT-MR images. This underlines the importance of registering CT to MRI for patients with oropharyngeal cancer, rather than viewing them separately. It also highlights the difficulty in delineating oropharyngeal cancers with CT due to the similarity in Hounsfield Units of tumour and surrounding tissue as well as artefacts caused by dental amalgam. While there was no significant difference between the mean GTV_D_ and GTV_RT_, there were important differences in the VMAT plans, as discussed below. To achieve the clinical goal of reduced late toxicities and improved tumour control using dose escalation with tighter PTV margins, uncertainties in GTV delineation need to be minimized and our data suggest PP_RT_ offers the optimal of the three set-ups.

The results reveal that there are potentially clinically relevant improvements to the quality of the VMAT plans when using PP_RT_ rather than PP_C_ or PP_D_. This is demonstrated by the PTV dose coverage, PTV dose homogeneity and instances of unmet dose constraints by the OARs. Due to improved registration accuracy and MRI being the recommended imaging modality for soft tissue oropharyngeal cancers [4], it may be assumed that PTV_RT_ would be the reference PTV. To determine the dose received by the reference, PTV_RT_, we optimized for PTV_C_ and PTV_D_ while calculating the dose to PTV_RT_. When optimizing for PTV_RT_ it was shown that the mean DVH for PTV_D_ had poorer dose coverage than PTV_C_ (Figure 4). Despite the magnitude of the mean GTV_RT_ and GTV_D_ being similar there may be differences in the shape and location of the GTV using these patient set-ups which would result in the DVHs of Figure 4. Neither the PP_C_ nor the PP_D_ VMAT plans were able to meet the clinical dose constraints of D_99_ and D_95_ for the reference, PTV_RT_. Furthermore, it has been argued that tumour control probability can be considerably compromised by an inhomogeneous dose to the PTV [25]. It is therefore suboptimal to use PP_C_ or PP_D_ rather than PP_RT_ for RT planning of patients with oropharyngeal cancer.

Obtaining a further MRI in the RT position, rather than using the original diagnostic MRI, may place greater demands on increasingly stretched healthcare resources. However, this must be weighed against the potential advantages of improved image registration and, by consequence, superior target volume definition and dose coverage of the PTV, as these results have demonstrated. Our study suggests further research, particularly in correlating dosimetric investigations with clinical outcome data, would be warranted.

## Conclusions

When MRI scans are performed in the RT position, as opposed to using diagnostic MR images not obtained in RT position, there are significant improvements in the quality of CT-MR registration. This study has also shown that RT positioned MRI scans offer improvements in target definition, dose coverage and dose homogeneity, which could have significant implications for tumour control rates. To our knowledge, this is the first study in the literature to confirm these advantages.

## Abbreviations

BDS: Beam directional shell; CT: Computed tomography; CTV: Clinical target volume; CTV LR: Lower risk clinical target volume; DVH: Dose volume histograms; GTV: Gross tumour volume; GTVC: Gross tumour volume delineated using PP_C_; GTVD: Gross tumour volume delineated using PP_D_; GTVRT: Gross tumour volume delineated using PP_RT_; MRI: Magnetic resonance imaging; MRID: MRI with the patient in the standard diagnostic position; MRIRT: MRI with the patient in the radiotherapy position in an immobilization mask; OAR: Organ at risk; PET: Positron emission tomography; PPC: Patient position protocol with the CT and MRI_D_ on a separate console; PPD: Patient position protocol with the CT and MRI_D_ registered; PPRT: Patient position protocol with the CT and MRI_RT_ registered; PTV: Planning target volume; PTV: LR Lower risk planning target volume; PTVC: Planning target volume generated from GTV_C_; PTVD: Gross tumour volume generated from GTV_D_; PTVRT: Gross tumour volume generated from GTV_RT_; RT: Radiotherapy; VMAT: Volumetric modulated arc therapy.

## Competing interests

The authors declare they have no competing interests.

## Authors’ contributions

SH and JF conceived the study and participated in its design. SH and MT coordinated the study. SH generated the radiotherapy plans and analyzed the data, SH, MMc, JF and MG helped draft the manuscript, LMT delineated the volumes for the radiotherapy plans and the GTVs. DG, AJ, MR and CP delineated the GTVs. All authors read and approved the final manuscript.
